# Metabolic Syndrome-Related Features in Controlled and Resistant Hypertensive Subjects

**DOI:** 10.5935/abc.20180076

**Published:** 2018-07

**Authors:** Arthur Santa Catharina, Rodrigo Modolo, Alessandra Mileni Versuti Ritter, Andréa Rodrigues Sabbatini, Heno Ferreira Lopes, Heitor Moreno Junior, Ana Paula de Faria

**Affiliations:** 1 Faculdade de Ciências Médicas - Universidade Estadual de Campinas (UNICAMP), Campinas, SP - Brazil; 2 Instituto do Coração (InCor) - Faculdade de Medicina da Universidade de São Paulo, São Paulo, SP - Brazil

**Keywords:** Metabolic Syndrome / diagnosis, Cardiovascular Diseases / mortality, Cholesterol, Waist Circumference, Triglycerides

## Abstract

**Background:**

Metabolic syndrome (MetS) is widespread among hypertensive patients. Clinical features and potential biomarkers of MetS in the presence of hypertension and resistant hypertension (RHTN) represent a great area of interest for investigation.

**Objective:**

The purpose of this study was to evaluate the prevalence of MetS and the clinical features associated with it in resistant and mild to moderate hypertensives.

**Methods:**

This cross-sectional study included 236 patients, (i) 129 mild to moderate hypertensive patients and (ii) 107 patients with RHTN. We measured blood pressure (BP) and adipokines levels, and performed bioelectrical impedance analysis. Microalbuminuria (MA), cardiac hypertrophy and arterial stiffness were also assessed. The significance level of alpha = 0.05 was adopted.

**Results:**

We found a MetS prevalence of 73% in resistant and 60% in mild-to-moderate hypertensive patients. In a multiple regression analysis, MA (odds ratio = 8.51; p = 0.01), leptin/adiponectin ratio (LAR) (odds ratio = 4.13; p = 0.01) and RHTN (odds ratio = 3.75; p = 0.03) were independently associated with the presence of MetS apart from potential confounders.

**Conclusions:**

Our findings suggest that both resistant and controlled hypertensive subjects have a high prevalence of MetS. In addition, MetS-related metabolic derangements may cause early renal and hormonal changes. Finally, LAR may be useful as a reliable biomarker for identifying those hypertensive subjects who are at risk for developing MetS.

## Introduction

Metabolic syndrome (MetS) is a cluster of metabolic abnormalities that affects approximately a quarter of worldwide adult population, which makes it a serious public health challenge.^1^ Ever since the MetS was described in 1988,^2^ several scientific organizations have attempted to formulate a general definition for the syndrome. The National Cholesterol Education Program - Adult Treatment Panel III (NCEP-ATPIII) definition^3^ has become the most widely used definition, probably because it provides a relatively simple approach for diagnosing MetS with easily measurable risk factors.

The relationship between MetS and cardiovascular diseases (CVDs) is noteworthy.^4^ In the largest meta-analysis on the theme comprising nearly one million patients, MetS was associated with a 2-fold increase in risk of CVD, cardiovascular mortality, myocardial infarction and stroke, and a 1.5-fold increase in the risk of all-cause mortality.^4^

The negative prognostic impact of MetS was also observed in patients with hypertension.^5^ Studies have shown a high prevalence of hypertension-related asymptomatic organ damage in hypertensive patients with MetS, such as left ventricular hypertrophy (LVH), elevated urinary albumin excretion rate and arterial stiffness.^5^ The majority of these patients have shown a deregulated production of adipokines.^6^ Adiponectin, an adipokine with anti-atherogenic, insulin sensitization, lipid oxidation, and vasodilatation activities^7^ showed to be decreased in obese and subjects with essential and resistant^8^ hypertension (RHTN). In contrast, elevated leptin levels are associated with MetS, hypertension and atherosclerosis. On the other hand, there are few data regarding MetS, resistant hypertension and mild to moderate hypertension. Thus, this study aimed to evaluate the prevalence of MetS and the clinical features associated with MetS in resistant and mild to moderate hypertensive patients.

## Methods

### Study population

In this cross-sectional study, a convenience sample of 107 resistant and 129 mild to moderate hypertensive patients regularly followed at the Resistant Hypertension Outpatient Clinic and Hypertension Outpatient Clinic of the University of Campinas (Campinas, Brazil) were enrolled, and classified into those with MetS (n = 157) and without MetS (n = 79). Suitable subjects who agreed to participate in the study were screened for a 6-month period of clinical follow-up to exclude (i) secondary hypertension (pheochromocytoma, aortic coarctation, Cushing's or Conn's syndrome, renal artery stenosis and obstructive sleep apnea) and (ii) pseudoresistance hypertension, including poor medication adherence (verified by pill counts) and white coat hypertension (verified by ambulatory blood pressure monitoring-ABPM).

The diagnosis of "true" RHTN was done according to the 2008 American Heart Association Scientific Statement,^9^ the last guideline published which properly defines a condition as (1) high blood pressure (BP) levels despite the use of at least three antihypertensive agents of different classes or (2) controlled BP after the use of four or more drugs. Ideally, one of the three agents should be a diuretic and all agents should be prescribed at optimal doses. Mild to moderate hypertensive subjects (grade I and II hypertension) were defined in accordance to the 2013 European Society of Hypertension (ESH) guidelines,^10^ the last guideline on essential hypertension. Exclusion criteria were clinically-evident coronary artery disease or cerebrovascular disease, significant impaired renal or liver function, myocardial infarction and peripheral vascular disease.

### Diagnosis of MetS

Diagnosis of MetS was determined according to the criteria proposed by the NCEP-ATPIII revised in 2005,^3^ as the presence of at least three of the following criteria: (i) waist circumference (WC) ≥ 88 cm for women or ≥ 102 cm for men, (ii) HDL-cholesterol < 50 mg/dL for women or 40 mg/dL for men, (iii) triglycerides ≥ 150 mg/dL (or in current use of fibrate), (iv) cutoff BP values of ≥ 130/85 mmHg (or current antihypertensive treatment), and (v) fasting glucose ≥ 100 mg/dL (or current treatment for type 2 diabetes).

#### Bioelectrical impedance analysis (BIA)

Fat-free mass (FFM), fat mass (FM), total body water (TBW) and basal metabolic rate (BMR) were determined by BIA using the Bioimpedance Analyser 450 (Biodynamics Corporation, Seattle, USA). The measurements were performed after 4-hour period of fasting. Also, patients were instructed to avoid physical activity and smoking prior to the examination.

#### Office and Ambulatory BP measurements

Office systolic BP (SBP) and diastolic BP (DBP) were evaluated at approximately 08:00 a.m. in the right arm using a validated digital sphygmomanometer (HEM-907XL, OMRON Healthcare Inc., Bannockburn, IL, USA).

The 24-h ABPM measurements were performed with a validated automatic device (Spacelabs 90217, Spacelabs Inc, Redmon, WA, USA), and measurements were taken every 20min. Patients were instructed to maintain their usual daily activities and inform them in a personal diary. Both office and ambulatory BP measurements were performed according to 2013 ESH guidelines.^10^

#### Biochemical measurements

The laboratory exams analyzed were: fasting blood glucose (FBG), insulin, glycated hemoglobin (HbA1c), serum sodium and potassium, plasma cortisol, total cholesterol, low and high-density lipoprotein-cholesterol (LDLc and HDLc, respectively), triglycerides, urea, creatinine and renin. The values between 30 and 300 mg/g of urine albumin/creatinine ratio grouped the patients as having microalbuminuria (MA) for comparisons of early renal damage. Plasma concentrations of adiponectin and leptin (R&D Systems, Minneapolis, USA) were determined by ELISA and aldosterone (Immunotech SAS, Marseille, France) by chemiluminescence, according to the manufacturer's instructions.

#### Pulse wave velocity

Arterial stiffness was determined by pulse wave velocity (PWV), in meters per second (m/s), dividing the distance between the right carotid and femoral arteries by the pulse transit time through these two sites of interest. We used the Sphygmocor device (AtCor Medical, USA), synchronized with the electrocardiogram. We used the mean of two PWV values in the analyses, or the median of three consecutive readings if the difference between the two measurements was greater than 0.5 m/s. The patients were considered as having arterial rigidity if PWV ≥ 10 m/s, for comparisons of vascular damage.^11^

### Echocardiography

Left ventricular (LV) measurements were performed according to the recommendations of the American Society of Echocardiography using two-dimensional M-mode echocardiography.^12^ Examinations were performed by an echocardiography expert and reviewed by two blinded investigators, following standard technique, using a cardiovascular ultrasound machine (Siemens Acuson CV70, Munich, Bavaria, Germany) with a multi-frequency sector transducer (2-4 MHz). We calculated LV mass index (LVMI), and considered those with LVMI > 95 g/m^2^ (females) and > 115 g/m^2^ (males) as having left ventricular hypertrophy (LVH). The intraobserver and interobserver coefficients of variation were less than 9.5% for the LVMI.

### Statistical analyses

For continuous variables we calculated the mean and standard deviation or median (1st, 3rd quartiles), according to normal distribution, measured by the Kolmogorov-Smirnov test. We compared them using either unpaired Student´s t-test or Mann-Whitney test, according to distribution of data. Categorical variables were presented in absolute numbers and/or percentages and compared by chi-square test. A logistic regression model was applied to determine association of clinical variables with the presence of MetS, apart from potential confounders. All statistical tests were performed using SigmaPlot 12.5 version (Systat software, Inc.). A significance level of alpha = 0.05 was adopted.

## Results

Baseline characteristics of hypertensive subjects with and without MetS are shown in Table 1. We found a MetS prevalence of 66% in all hypertensive population. No differences were found between groups regarding age, race and gender. As expected, BMI, WC, FM and TBW were higher in hypertensive patients with MetS. Office heart rate (HR) was significantly higher in patients with MetS. Neither office and ambulatory BP levels nor the proportion of patients with uncontrolled office BP (≥ 140/90 mmHg) were different between groups. The patients with MetS showed a higher prevalence of MA compared to their counterparts. The medication use was similar between groups, except for the calcium channel blockers and antidiabetics that were higher in MetS group (Table 1).

**Table 1:** General characteristics of hypertensive patients with and without metabolic syndrome

	Patients with MetS (n = 157)	Patients without MetS (n = 79)	p-value
**Clinical data**			
Age (years)	63 (56 – 70)	65 (56 – 71)	0.39
White race (%)	122 (77)	52 (65)	0.05
Female gender (%)	106 (67)	47 (59)	0.23
BMI (kg/m^2^)	31 (27 – 34)	26 (23 – 28)	< 0.01
WC (cm)	100 ± 13	89 ± 12	< 0.01
FFM (Kg)	54 (46 – 62)	52 (44 – 63)	0.13
FM (Kg)	24 (19 – 31)	17 (13 – 23)	< 0.01
TBW (%)	74 (72 – 75)	73 (72 – 75)	0.03
BMR (cal/day)	1672 (1436 – 1947)	1616 (1369 – 1954)	0.23
Office SBP(mmHg)	142 (134 – 150)	146 (132 – 154)	0.39
Office DBP(mmHg)	82 (75 – 89)	82 (80 – 88)	0.44
Office HR (bpm)	67 (61 – 76)	64 (58 – 72)	0.01
24h-ABPM SBP(mmHg)	128 (118 – 139)	129 (118 – 136)	0.78
24h-ABPM DBP(mmHg)	77(70 – 81)	78 (70 – 86)	0.28
ABPM HR (bpm)	64 ± 14	64 ± 13	0.94
Uncontrolled office BP (%)	96 (61)	48 (60)	0.97
**TODs**			
MA ≥ 30 (mg.g^-1^), n (%)	31 (20)	3 (4)	< 0.01
PWV ≥ 10 (m.s^-1^), n (%)	68 (43)	35 (44)	0.94
LVH, n (%)	83 (53)	44 (55)	0.96
**Medication**			
Total anti-HA drugs	3 (2 – 4)	3 (2 – 4)	0.27
Diuretics, n (%)	123 (78)	64 (80)	0.75
CCBs, n (%)	112 (71)	42 (52)	< 0.01
ACEIs, n (%)	36 (22)	26 (32)	0.13
ARAs, n (%)	108 (69)	48 (60)	0.27
Beta-blockers, n (%)	67 (43)	28 (35)	0.39
Spironolactone, n (%)	33 (21)	8 (10)	0.06
Central α-agonists, n (%)	24 (15)	8 (10)	0.37
Oral antidiabetics, n (%)	90 (57)	16 (20)	< 0.01
Statins, n (%)	111 (70)	51 (63)	0.41
Antiplatelet drugs, n (%)	67 (43)	23 (29)	0.06

Values are expressed as mean ± standard deviation or median (1st, 3rd quartiles), according to data distribution. Continuous variables were compared using unpaired Student´s t-test or Mann-Whitney test, according to data distribution. Categorical variables were compared by chi-square test. BMI: body mass index; WC: waist circumference; FFM: fat free mass; FM: fat mass; TBW: total body water; BMR: basal metabolic rate; SBP: systolic blood pressure; DBP: diastolic blood pressure; HR: heart rate; ABPM: ambulatory blood pressure monitoring; LVH: left ventricular hypertrophy; MA: microalbuminuria; PWV: pulse wave velocity; CCBs: calcium channel blockers; ACEIs: angiotensin converting enzyme inhibitors; ARAs: angiotensin II receptor antagonist; TODs: target organ damages.

As expected, the evaluation of biochemical parameters showed increased triglycerides, as well as fasting glucose and HbA1c in subjects with MetS (Table 2). Additionally, adiponectin levels were significantly lower in patients with MetS, while leptin demonstrated to be increased in those patients, compared to the subjects without MetS (Table 2).

**Table 2:** Biochemical parameters of hypertensive patients with and without metabolic syndrome

	Patients with MetS (n = 157)	Patients without MetS (n = 79)	p-value
Cholesterol (mg.dL^-1^)	166 (139 – 192)	179 (150 – 200)	0.06
LDL-c (mg.dL^-1^)	88 (70 – 111)	98 (73 – 118)	0.19
HDL-c (mg.dL^-1^)	43 (37 – 49)	57 (51 – 65)	< 0.01
Triglycerides (mg.dL^-1^)	142 (97 – 199)	81 (68 – 115)	< 0.01
FBG (mg.dL^-1^)	107 (95 – 130)	91 (86 – 97)	< 0.01
HbA1c (%)	6.30 (6– 7.40)	5.90 (5.50 – 6)	< 0.01
hs-CRP (mg.dL^-1^)	0.39 (0.17 – 0.65)	0.25 (0.11 – 0.48)	0.02
Na (mEq.dL^-1^)	141 (140 – 143)	142 (138 – 143)	0.61
K (mEq.dL^-1^)	4.40 (4.10 – 4.70)	4.30 (4.20 – 4.60)	0.82
PAC (ng.dL^-1^)	83 (48 – 162)	65 (41 – 125)	0.10
CC (ml.min^-1^.(1,73m^2^)^-1^)	80 (55 – 97)	71 (53 – 94)	0.53
Creatinine (mg.dL^-1^)	0.93 (0.80 – 1.12)	0.95 (0.77 – 1.20)	0.97
Renin (pg.ml^-1^)	23 (12 – 64)	30 (11 – 80)	0.78
Urea (mg.mL^-1^)	35 (26 – 44)	36 (28 – 44)	0.81
Cortisol (ug.dL^-1^)	14 (10 – 20)	14 (10 – 16)	0.44
Leptin (ng.mL^-1^)	21.0 (14.40–41.60)	15.70 (6.30–33.20)	< 0.01
Adiponectin (µg.dL^-1^)	5.30 (2.60– 7.80)	7.50 (3.80 – 11.90)	< 0.01
LAR	4.81 (2.14 – 10.80)	2.22 (1.10 – 5.20)	< 0.01
LAR > 3.72, n (%)	85 (54)	24 (30)	< 0.01

Values are expressed as mean ± standard deviation or median (1st, 3rd quartiles), according to data distribution. Continuous variables were compared using unpaired Student´s t-test or Mann-Whitney test, according to data distribution. Categorical variables were compared by chi-square test. MetS: metabolic syndrome; LDL-c: low density lipoprotein-c; HDL-c: high density lipoprotein-c; FBG: fasting blood glucose; HbA1C: glycated hemoglobin; hs-CRP: high-sensitivity c-reactive protein; Na: serum sodium; K: serum potassium; PAC: plasma aldosterone concentration; CC: creatinine clearance; LAR > 3.7: leptin adiponectin ratio > 3.7 (the cutoff value was determined by median value).

Finally, the multiple logistic regression revealed that MA, leptin/adiponectin ratio (LAR) and resistance to antihypertensive treatment were independently associated with the presence of MetS (Table 3).

**Table 3:** Multiple logistic regression for the presence of metabolic syndrome*

	Odds ratio	95% CI	p-value
LAR > 3.7	4.13	1.38 – 12.34	0.01
HR (bpm)	0.97	0.92 – 1.03	0.39
MA > 30 (mg.g^-1^)	8.51	1.53 – 47.14	0.01
hs-CRP (mg.dL^-1^)	2.92	0.83 – 10.19	0.09
RHTN	3.75	1.09 – 12.92	0.03

* The variables in this model were also adjusted for age, gender and race. MetS: metabolic syndrome; hs-CRP: high-sensitivity c-reactive protein; HR: heart rate; MA: microalbuminuria; RHTN: resistant hypertension; LAR > 3.7: leptin adiponectin ratio > 3.7 (the cutoff value was determined by median value).

## Discussion

Our main findings suggest that MA and increased LAR are associated with the presence of MetS in hypertensive population, apart from potential confounders. Also, resistance to antihypertensive treatment is strongly associated with MetS. The high prevalence of these coexisting conditions - hypertension and MetS - may explain the increased prevalence of hypertension-related target organ damage (TOD), such as elevated urinary albumin excretion.^5^ Additionally, this early renal organ damage may in part explain the increased cardiovascular risk conferred by MetS in hypertensive patients, since this marker of TOD is a well-known predictor of CV events.^13^ In this sense, the identification and treatment of risk factors for cardiovascular and renal diseases, as well as an early detection of hypertension-related TOD may directly affect the prognosis of hypertensive patients with MetS.^14^

Our finding of increased MA in hypertensive patients with MetS is supported by previous studies.^13^ The common underlying mechanisms that may explain increased MA in patients with MetS include factors such as: (i) overactivation of the renin-angiotensin system; (ii) increase in oxidative stress and (iii) inflammation.^15^ In addition, the presence of MA may affect reflect progressive endothelial and vascular dysfunction.^16^ It is worth to mention that we found no difference in BP levels between the groups. Thus, in our cross-sectional study MA is probably associated with other components that comprise MetS. Another hypothesis is that the greater use of calcium channel blockers by hypertensive patients with MetS could have resulted in BP control, but not in avoiding early renal damage, in agreement with several studies.^17^ Another point to be mentioned is that despite of the greater use of antidiabetic drugs by patients with MetS, HbA1c remained higher in this group. On the other hand, studies^18^ have consistently shown that levels of HbA1c < 7% are associated with a reduced risk of structural and clinical manifestations of diabetic nephropathy in patients with diabetes type 1 and type 2. For instance, the U.K. Prospective Diabetes Study (UKPDS)^18^ demonstrated a nearly 30% risk reduction for the development of MA in the group intensively treated for hyperglycemia (HbA1c of 7%).^18^

Hypoadiponectinemia and hyperleptinemia are commonly found in hypertensive and obese patients. Previous studies have shown an inverse association between adiponectin levels and low-grade albuminuria in essential^19^ and resistant hypertensive patients.^20,21^ Similarly in experimental studies, adiponectin knockout rats have higher levels of albuminuria (twice above normal values), and after replacement of the protein, albuminuria returned to its normal levels.^22^ Hyperleptinemia is also an independent risk factor for coronary artery disease ^23^ and strong predictor of acute myocardial infarction. Besides that, leptin acts as a powerful sympathostimulator, associated with increased BP and tachycardia, which consequently contributes to obesity-related hypertension and kidney damage.^24^ Furthermore, a study has supported that the LAR is more beneficial than either alone for the diagnosis of MetS.^25^ The use of LAR has the potential to assess insulin sensitivity and MetS in the non-fasting state, since the difference between adiponectin and leptin tends to be small in the fasting *versus* postprandial state.^26^ Our study showed that LAR was independently associated with the presence of MetS. There are several studies that relate MetS to various cytokines and adipokines, but no biomarker is currently used in clinical practice to help in predicting and establishing MetS in individuals. Therefore, the deregulated adipokine levels (LAR) might be a valuable tool for diagnosis, prognosis or even early detection of MetS in the high-risk hypertensive population, although these associations should be tested. This may also guide a rational therapeutic approach and risk management, since adipokines are altered after lifestyle modifications and medications.^27,28^

**Figure 1:**
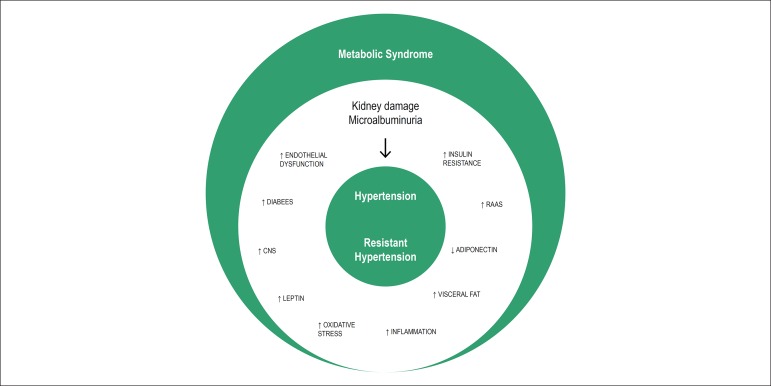
Diagrammatic representation of the metabolic syndrome effects on hypertension and resistant hypertension (RHTN). Abbreviations: renin-angiotensin-aldosterone system (RAAS); central nervous system (CNS).

The prevalence of MetS has been increasing worldwide,^29^ and it is higher in hypertensive patients than in general population.^5^ In our study, we found a considerable prevalence of MetS in all hypertensive subjects (66%) - 73% in resistant and 60% in mild-to-moderate hypertensive patients. Similar data have been reported in the Global Cardiometabolic Risk Profile in Patients with hypertension disease (GOOD) study,^30^ in which 58% of essential hypertensive patients had MetS. Indeed, other similar study also indicated a high proportion of RHTN among patients with MetS.^31^ This high prevalence may be explained by the older age of the population in the studies, since prevalence of MetS is highly age-dependent.^1^ In our study, RHTN was associated with MetS independently of potential confounders. Although our study does not affirm causality between this association, it seems reasonable to say that the metabolic derangements associated with MetS promote alterations in the vasculature and the kidney that might lead to RHTN and chronic kidney disease.^32^ Furthermore, the increased renal impairment in patients with MetS is probably linked to the underlying condition of prior hypertension in these patients^33^ (Figure 1). In this context, our findings highlighted the importance of improving strategies to prevent cardiovascular and renal outcomes. Still, it points out that not only RHTN patients require a close clinical attention, but also mild to moderate hypertensive subjects, who demonstrated a high prevalence of MetS comparable to RHTN patients.

Finally, pharmacological approaches should be carried out in order to improve obesity, dyslipidemia, hyperglycemia and hypertension^33^ for renal protection. However, the cornerstone of treating MetS remains lifestyle modification,^3^ which mainly involves healthy diet, aerobic exercise, and behavioral counseling. To date, current guidelines do not specifically address the management of mild to moderate hypertension and RHTN in the patient with MetS. However, considering the increased risk of developing diabetes in these patients, it seems reasonable that the first consideration in antihypertensive treatment is to be focused on the inhibition of the renin-angiotensin system with either angiotensin converting enzyme or angiotensin II receptor inhibitors.^34^ There has been increasing interest in combination strategies of antihypertensive agents in RHTN patients with MetS to reduce the pill burden. Future works are still needed to define the best antihypertensive therapy in this group of high-risk patients.

The limitations of this study include: (i) the cross-sectional design with no cause-effect inference; (ii) a small sample size and (iii) inclusion of patients from one outpatient clinic only. Although studies have shown significant differences between patients with mild to moderate hypertension and RHTN,^35,36^ we did not dichotomize the hypertensive population because they both had a high prevalence of SMet with similar metabolic profile, then contributing to the objective of evaluating the influence of SMet on all these subjects together.

## Conclusion

In summary, our study showed that MetS is significantly associated with MA, RHTN and adipokines levels. These findings suggest that hypertensive patients with MetS tend to develop early manifestations of end-organ damage with metabolic/hormonal changes, culminating in increased cardiovascular risk and renal impairment. However, as we mentioned earlier, we cannot infer from this cross-sectional study the exact nature of the association between MetS, MA, RHTN and adipokines levels. Early diagnosis of MetS in hypertensive patients may enable more accurate prediction of adverse cardiovascular events and renal impairment, as well as the implementation of more efficient strategies in terms of primary prevention. Besides that, prompt identification of MetS in resistant hypertensive patients allows modification of multiple risk factors that promote resistance to antihypertensive therapy, as well as guide the treatment to individual components of the syndrome. Thus, targeted treatment to individual components of the syndrome along with weight loss and lifestyle modifications can prevent resistance to antihypertensive treatment, as well as contribute to effective therapy in resistant hypertensive patients with MetS. Given the alterations that MetS confers on RHTN, future clinical trials can begin to address this important topic. Once the syndrome is identified, lifestyle changes and a different therapeutic approach can enhance the prognosis of the disease. Indeed, further studies on LAR in a larger hypertensive population with MetS is needed to assess whether this marker is sensitive and specific for identifying those who are at risk for developing MetS. The LAR could be used as a relatively easy, minimally-invasive tool for early MetS diagnosis and, consequently, decrease the chance of maladaptive effects caused by this syndrome.
